# Pharmacological interventions for co-occurring psychopathology in people with borderline personality disorder: secondary analysis of the Cochrane systematic review with meta-analyses

**DOI:** 10.1192/bjp.2024.172

**Published:** 2025-04

**Authors:** Johanne Pereira Ribeiro, Sophie Juul, Mickey T. Kongerslev, Mie Sedoc Jørgensen, Birgit A Völlm, Henriette Edemann-Callesen, Christian Sales, Julie P. Schaug, Klaus Lieb, Erik Simonsen, Jutta M. Stoffers-Winterling, Ole Jakob Storebø

**Affiliations:** Center for Evidence-Based Psychiatry, Psychiatric Research Unit, Psychiatry Region Zealand, Slagelse, Denmark; and Department of Psychology, University of Southern Denmark, Odense, Denmark; Copenhagen Trial Unit, Centre for Clinical Intervention Research, The Capital Region of Denmark, Copenhagen University Hospital – Rigshospitalet, Copenhagen, Denmark; and Stolpegaard Psychotherapy Centre, Mental Health Services in the Capital Region of Denmark, Copenhagen, Denmark; Department of Psychology, University of Southern Denmark, Odense, Denmark; and Mental Health Services West, Psychiatry Region Zealand, Slagelse, Denmark; Center for Evidence-Based Psychiatry, Psychiatric Research Unit, Psychiatry Region Zealand, Slagelse, Denmark; Department for Forensic Psychiatry, University Medicine Rostock, Rostock, Germany; Nottinghamshire Healthcare NHS Foundation Trust, Nottingham, UK; Department of Psychiatry and Psychotherapy, University Medical Center Mainz, Mainz, Germany; Mental Health Services East, Copenhagen University Hospital, Psychiatry Region Zealand, Copenhagen, Denmark; and Department of Clinical Medicine, Faculty of Health and Medical Sciences, University of Copenhagen, Copenhagen, Denmark

**Keywords:** Comorbidity, pharmaceutical drug trial, clinical trials, mental health services, personality disorders

## Abstract

**Background:**

Medications are commonly used to treat co-occurring psychopathology in persons with borderline personality disorder (BPD)

**Aims:**

To systematically review and integrate the evidence of medications for treatment of co-occurring psychopathology in people with BPD, and explore the role of comorbidities.

**Method:**

Building on the current Cochrane review of medications in BPD, an update literature search was done in March 2024. We followed the methods of this Cochrane review, but scrutinised all identified placebo-controlled trials *post hoc* for reporting of non BPD-specific (‘co-occurring’) psychopathology, and explored treatment effects in subgroups of samples with and without defined co-occurring disorders. GRADE ratings were done to assess the evidence certainty.

**Results:**

Twenty-two trials were available for quantitative analyses. For antipsychotics, we found very-low-certainty evidence (VLCE) of an effect on depressive symptoms (standardised mean difference (SMD) −0.22, *P* = 0.04), and low-certainty evidence (LCE) of an effect on psychotic–dissociative symptoms (SMD −0.28, *P* = 0.007). There was evidence of effects of anticonvulsants on depressive (SMD −0.44, *P* = 0.02; LCE) and anxious symptoms (SMD −1.11, *P* < 0.00001; VLCE). For antidepressants, no significant findings were observed (VLCE). Exploratory subgroup analyses indicated a greater effect of antipsychotics in samples including participants with co-occurring substance use disorders on psychotic–dissociative symptoms (*P* = 0.001).

**Conclusions:**

Our findings, based on VLCE and LCE only, do not support the use of pharmacological interventions in people with BPD to target co-occurring psychopathology. Overall, the current evidence does not support differential treatment effects in persons with versus without defined comorbidities. Medications should be used cautiously to target co-occurring psychopathology.

Borderline personality disorder (BPD) commonly co-occurs with other mental health disorders. Large epidemiological studies report prevalence rates of around 80% for confirmed diagnoses of mood disorders and substance use disorders (SUDs).^1^ Other disorders are also highly prevalent, such as post-traumatic stress disorder (PTSD), eating disorders and attention-deficit hyperactivity disorder.^2,3^ However, methodologically high-standing clinical trials, such as randomised controlled trials (RCTs), often use highly selected samples that explicitly exclude these co-occurring diorders.^4,5^ Although psychotherapy research has advanced in terms of developing and evaluating adapted treatments designed for patients who have defined comorbidities such as PTSD, SUDs or eating disorders, to our knowledge, no placebo-controlled RCTs have tested the effects of major pharmacological intervention classes (i.e. antipsychotics, antidepressants or anticonvulsants) used in BPD samples to treat co-occurring psychopathology.

## The disconnection between clinical guidelines and practice

Despite international guidelines consistently advising against the use of pharmacological interventions for continued BPD treatment,^6–9^ impulsivity, affective instability and dissociative symptoms are still considered targets of psychopharmacological treatments in clinical practice.^10,11^ Consequently, medication rates are high in clinical settings, and although a decrease in overall medication use can be observed, co-occurring psychopathology remain the main factor for the prescription of pharmacological interventions, as well as for polypharmacy.^10^ Although co-existing conditions such as major depressive disorder (MDD) may require pharmacological intervention, and guidelines advise to apply to the clinical guidelines for these co-occurring disorders,^7,8^ the evidence regarding these interventions is very limited. To our knowledge, there are no placebo-controlled RCTs testing the effects of pharmacological interventions in samples of people with a diagnosis of BPD plus another confirmed co-occurring psychopathology, despite the high rates of co-occurring psychopathology in this population. Instead, the generalisability of clinical trials in the field of BPD has been questioned by a study that scrutinised participants of a large epidemiological study, regarding their eligibility to enter pharmacological clinical trials, and found that as many as 76% of community-dwelling people with BPD would have been excluded from such trials.^4^

## Recent findings: insights from a Cochrane systematic review

We recently published an update of a Cochrane systematic review on pharmacological interventions for people with BPD.^12^ It included 46 RCTs that comprised 2769 participants with a confirmed diagnosis of BPD. No restrictions were applied concerning co-occurring psychopathology. The main intervention categories were antipsychotics, antidepressants and anticonvulsants. Primary outcomes comprised BPD symptom severity, self-harm, suicide-related outcomes and psychosocial functioning. Secondary outcomes comprised specific BPD symptoms as defined by the individual nine DSM-5 criteria,^13^ attrition and adverse events.

Following up on the Cochrane review, we assessed the following clinical questions in this publication: (a) What are the effects of pharmacological interventions compared with placebo in people with BPD on common co-occurring psychopathology not specific to BPD (e.g. depressive, anxious, dissociative, substance use or eating disorder symptoms)? and (b) Is there any evidence of differential outcomes depending on the type of co-occurring psychopathology in samples including participants with defined co-occurring psychopathology (e.g. MDD, SUD, PTSD) versus samples excluding them?

## Method

This systematic review follows the Preferred Reporting Items for Systematic Reviews and Meta-Analyses (PRISMA) guidelines (see PRISMA checklist, Supplementary Appendix 1 available at https://doi.org/10.1192/bjp.2024.172) and is associated with our Cochrane systematic review ‘Pharmacological interventions for people with borderline personality disorder’.^12^ The protocol was preregistered in PROSPERO (identifier CRD42018091044) and published in the Cochrane Library in 2018.^14^

This paper has two main aims. The first aim was to explore the effects of medications on co-occurring psychopathology in persons with BPD. As co-occurring psychopathology, we defined psychopathology outcomes that are not defined as BPD criteria by the DSM-5.^15^ In the 2022 Cochrane review, such outcomes were limited to depressive and psychotic symptoms. Therefore, all included placebo-controlled trials were re-assessed for any additional outcomes regarding symptoms of co-occurring disorders at the end of treatment (such as symptoms of PTSD, anxiety, psychosis, depression, substance misuse, eating disorder, etc.), and analysed *ad hoc*.

The second aim was to explore if there is reason to assume that medication effects on co-occurring psychopathology might differ depending on the co-occurrence of manifest psychiatric comorbidities. Therefore, the placebo-controlled effects of medications on co-occurring psychopathology in selected versus non-selected subgroups were compared in exploratory subgroup analyses *post hoc*, using the individual trial eligibility criteria for specific co-occurring psychiatric disorders (i.e. whether trials excluded specific co-occurring psychiatric disorders or not).

We applied these *post hoc* exploratory subgroup analyses to all outcomes of co-occurring psychopathology. As co-occurring psychopathology outcomes, we used those outcomes that were predefined in the Cochrane 2022 review (i.e. depressive and dissociative symptoms), as well as those outcomes of co-occurring psychopathology that we identified *ad hoc* in the primary studies. We deemed a minimum of three effect sizes per comparison and outcome necessary for combing them meta-analytically.

### Search strategy

The full methodology of this systematic review is predefined and described elsewhere.^12^ In short, we included RCTs that investigated any pharmacological interventions compared with placebo for people with a BPD diagnosis according to any available version of the DSM and ICD. We included trials if at least 70% of participants had a BPD diagnosis. If a smaller proportion of trial participants had a BPD diagnosis, we contacted the study authors and requested data for the BPD subsample. Trials with or without co-occurring psychopathology were included.

For the Cochrane review, we searched 21 databases and trial registries until 21 February 2022. This search was updated on 6 March 2024 in Medline, Cochrane Central, EMBASE, PsycINFO, CINAHL, Web of Science, ERIC, LILACS, ProQuest Dissertations and Sociological Abstracts. In addition, we traced all studies categorised as ‘ongoing’ in the full review,^12^ for meantime end-point data publications. Two independent review authors conducted title and abstract screening and full-text screening. A third review author settled any discrepancies. We additionally traced cross-references from relevant literature and contacted trial authors and sponsors regarding any unpublished data.

### Data extraction and critical assessment of included trials and evidence summaries

Data extraction was carried out by two independent review authors (J.P.R. and S.J.), using a predefined data extraction template for consistency. Data extraction was done starting 1 June 2022 for the full Cochrane review, and starting 15 April 2024 for the update search. Additionally, we specifically searched for any end-of-treatment data on co-occurring psychopathology. In each included trial, we extracted all available information on inclusion and exclusion criteria, as well as the baseline data on co-occurring psychopathology.

We assessed the risk of bias in individual trials by using the Cochrane Collaboration Risk of Bias tool (RoB). Since assessments took place in the context of the full Cochrane review, which was started before publication of the current Cochrane RoB-2 tool, all assessments are based on the first version of the RoB tool.^16^ All risk-of-bias assessments were conducted independently by two authors, and discrepancies were settled by a third review author.

In accordance with Cochrane standards, the Grading of Recommendations, Assessment, Development and Evaluations (GRADE) tool^17–20^ was used to assess the quality, i.e. certainty, of the statistically integrated evidence. Using GRADE, the body of evidence for each comparison and outcome (i.e. the pooled effect estimates) is categorised as high, moderate, low or very low certainty. We considered the following factors as suggested by GRADE: risk of bias in primary studies (based on risk-of-bias ratings previously conducted by use of the Cochrane RoB tool), inconsistency, indirectness and imprecision. Moreover, funnel plots were drawn to examine publication bias if at least ten effect estimates were available.^16^ Two authors (J.P.R. and J.M.S.-W.) independently conducted all GRADE assessments and solved any discrepancies by discussion.^17–20^

### Data analysis

We included data on intention-to-treat samples whenever possible. If diverse measurement scales were used to assess the same outcome in primary studies, we calculated standardised mean differences (SMDs) and corresponding 95% confidence intervals. Estimates for continuous outcomes were generated with inverse variance. SMD summary estimates were generated with random-effects models, as we expected substantial heterogeneity among trials. Heterogeneity was assessed by use of the *I*^2^ score.^21^ As inconsistency depends also on additional factors such as the magnitude and direction of effects, or *χ*^2^ *P*-values, we considered both the *I*^2^ statistic and visual inspection of the forest plot and exclusion of outliers to assess heterogeneity, instead of solely relying on the *I*^2^ statistic. According to the Cochrane handbook, heterogeneity was therefore considered moderate when between 30 and 60%, substantial when between 50 and 90%, and considerable when between 75 and 100%.^22^ For predefined methods not used in this review, see Supplementary Appendix 2.

We conducted sensitivity analyses in terms of excluding outliers, defined as individual study estimates for which the 95% confidence interval boundaries did not overlap with the 95% confidence interval of the pooled effect estimates.^23^ As in the main review, we also performed sensitivity analyses in terms of excluding (primary analyses) versus including (secondary analyses) data from cross-over trials for which no separate data for the period before first cross-over were available, using end-of-period data.^24,25^

To examine the role of comorbid psychopathology in pharmacotherapy effects, we conducted subgroup analyses to compare samples excluding versus not excluding defined psychiatric comorbidities. Such subgroup analyses were only done if, for each subgroup, a minimum of three primary studies was available.

Analyses along with forest plots and risk-of-bias tables were generated with RevMan5 version 5.4.1 for Windows (The Cochrane Collaboration, London, UK; see https://training.cochrane.org/online-learning/core-software/revman).^26^

## Results

### General description of all included trials

**Fig. 1:**
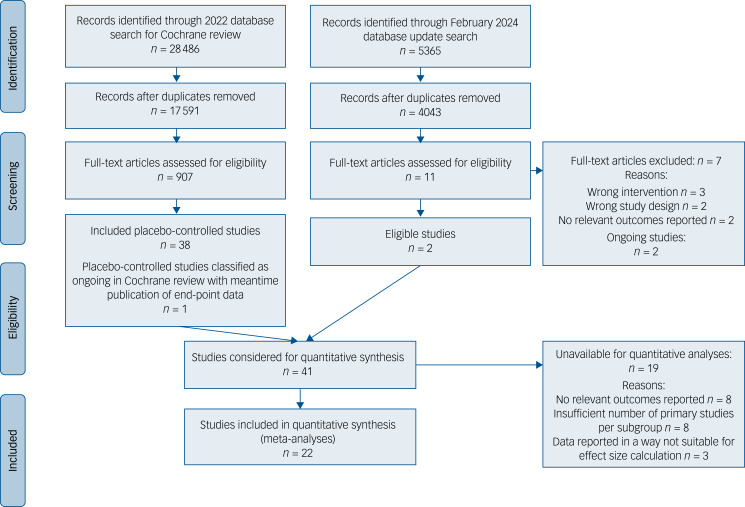
Preferred Reporting Items for Systematic Reviews and Meta-Analyses flow chart of study selection.

Of the 46 trials included in the Cochrane review, 38 had a placebo group and were considered for quantitative analyses here. In March 2024, an update search plus tracking of trials included as ongoing in the 2022 Cochrane review yielded three more eligible, placebo-controlled trials with published end-point data.^27–29^ Therefore, 41 placebo-controlled trials were considered for inclusion into quantitative analyses (Fig. 1).

Nineteen of the 41 trials were not included in the quantitative analyses for the following reasons: eight trials provided no outcomes relevant to this review,^27,30–36^ another eight trials^29,36–40^ were not included because of a lack of a sufficient number of trials testing the same kind of substance (minimum requirement: three poolable effect estimates per analysis), and three trials reported data in a way that was not suitable for effect size calculations.^41–43^ Study characteristics of these trials are detailed in Supplementary Appendix 3. After exclusion of these trials, 22 trials were included in the quantitative analyses (Fig. 1, Supplementary Appendix 4).

Characteristics of the included trials are detailed in Table 1. Altogether, the 22 trials comprised 1690 participants with BPD. The mean age ranged from 21.7^63^ to 40.7 years.^64^ Five trials included females only;^46,49,58,60,62^ all remaining trials included both genders, but with predominantly females.

**Table 1:** Characteristics of trials available for quantitative analyses (22 randomised controlled trials, reporting on 26 placebo-controlled comparisons)

Study	Country	Gender (% female)	Age (years), mean	Sample population comorbidity	Concomitant medication allowed	Setting	Design	Duration	Sample size	Intervention	Comparator	Outcomes relevant to this review
Antipsychotics versus placebo												
Black et al^44^	USA	70.53	29.5	Mood disorders, anxiety disorders, substance misuse and other Axis 1 disorders	Benzodiazepines and anticholinergics were allowed	Out-patient	Parallel	8 weeks	95	Quetiapine	Placebo	Depressive symptoms
Bogenschutz and Nurnberg^45^	USA	62.50	32.6	Mean of 2.9 SCID-II personality disorders (including BPD) and a mean of 2.2 Axis 1 diagnoses	Medication for stable, chronic medical conditions such as hypertension	Out-patient	Parallel	12 weeks	40	Olanzapine (2.5–20 mg/d), most 5–10 mg/d, mean at end-point 6.9 mg/d (s.d. 3.2)	Placebo	Anxiety symptoms Psychotic symptoms
Cowdry and Gardner^46^	USA	100	31.6	Other Axis 2 personality disorders (dependent, avoidant, histrionic schizotypal, narcissistic and paranoid), and Axis 1 disorders (atypical bipolar disorder, major depressive episode with and without melancholia, dysthymia)	Unclear, no information	Out-patient	Cross-over, multi-arm	6 weeks	16^c^	Trifluoperazine hydrochloride	Placebo	Depressive symptoms Anxiety symptoms
Crawford et al^28^	UK	76%	30.0	High rates of personality disorder comorbidity (90% were rated as having a moderate or severe personality disorder (SASPD criteria))	No restrictions on the use of other treatments	In-patient (forensic setting)	Parallel	12 weeks	29	Clonidine	Placebo	Dissociation/psychotic-like symptoms
Goldberg et al^47^	USA	58	32	Schizotypal personality disorder^a^	Unclear, no information	Out-patient	Parallel	12 weeks	40	Thiothixene	Placebo	Depressive symptoms Anxiety symptoms Psychotic symptoms
Grant et al^48^	USA	53.6	39.7	Anxiety disorder, mood disorder, eating disorder, ADHD	Allowed as prescribed (antidepressants, mood stabilisers and stimulants)	Out-patient	Parallel	12 weeks	80	Brexpiprazole	Placebo	Depressive symptoms Anxiety symptoms
Linehan et al^49^	USA	100	36.8	Unclear, no information	Unclear, no information	Out-patient	Parallel	24 weeks	24	Olanzapine (2.5–15 mg/d, mean final dose 4.46, s.d. 1.16) plus DBT	Placebo plus DBT	Depressive symptoms
Nickel et al^50^	Germany	82.69	21.65	Depressive disorders, anxiety disorders, obsessive–compulsive disorders and somatoform disorders	Not allowed	Out-patient	Parallel	8 weeks	52	Aripiprazole	Placebo	Depressive symptoms Anxiety symptoms Psychotic symptoms
Pascual et al^51^	Spain	81.67	29.2	Unclear, there is no information on population comorbidity. However, many psychiatric disorders were excluded	It was allowed to continue with benzodiazepine (maximum of 40 mg/d), antidepressants and mood stabilisers if initiated before inclusion; doses could not be modified	Out-patient	Parallel	12 weeks	60	Ziprasidone	Placebo	Depressive symptoms Anxiety symptoms Psychotic symptoms
Schulz et al^65^	Belgium, France, Germany, Norway, Portugal, Spain, Sweden, UK and USA	71.02	31.81	No apparent comorbidity (based on exclusion criteria)	No medications with primarily CNS activity (except for protocol-specified benzodiazepines and hypnotics)	Out-patient	Parallel	12 weeks	314	Olanzapine (2.5–20 mg/d, mean final dose 7.09 mg/d, s.d. 5.11)	Placebo	Depressive symptoms Psychotic symptoms
Soler et al^52^	Spain	86.57	40.74	No apparent comorbidity, (e.g. patients with unstable comorbid Axis 1 disorders were excluded)	Treatment with benzodiazepines, antidepressants and mood stabilisers could be continued, but doses could not be modified during the trial	Out-patient	Parallel	12 weeks	60	Olanzapine plus DBT	Placebo plus DBT	Depressive symptoms Anxiety symptoms
Soloff et al^53^	USA	75.56	25.1	Comorbid schizotypal personality disorder^^^^	Biperiden hydrochloride (2 mg)	In-patient	Parallel, multi-arm	5 weeks	90	Haloperidol (amitriptyline)	Placebo	Anxiety Depression Dissociation/psychosis
Cornelius et al^54^	USA	75.93	26.7	Major depressive disorder	Not allowed	In- and out-patients	Parallel, multi-arm	5 weeks	108	Haloperidol (phenelzine sulphate)	Placebo	Depression Anxiety Dissociation/psychosis
Zanarini et al^66^	Argentina, Chile, Italy, Peru, Poland, Romania, Turkey, USA and Venezuela	73.61	32.98	Mood disorders, substance use disorders, anxiety disorders, eating disorders and Axis 2 disorders besides BPD	Benzodiazepines, hypnotics, lorazepam and episodic use of anticholinergics, benztropine mesylate, biperiden or trihexyphenidyl allowed during trial at specific doses; however, prophylactic use of anticholinergics was not allowed	Out-patient	Parallel	12 weeks	301^b^	Olanzapine (5–10 mg/d, mean dose 6.66 mg/d)	Placebo	Depression Dissociation/psychosis
Ziegenhorn et al^55^^c^	Germany	94.44	32	88% had comorbid disorders: PTSD, eating disorders and/or substance misuse	Stable psychotropic medication regimen of up to three psychotropic drugs allowed	In-patient	Cross-over	18 weeks	18	Clonidine	Placebo	Depression Dissociation/psychosis
Anticonvulsants versus placebo												
Cowdry and Gardner^46^	USA	100	31.6	Other Axis 2 personality disorders (dependent, avoidant, histrionic schizotypal, narcissistic and paranoid), and Axis 1 disorders (atypical bipolar disorder, major depressive episode with and without melancholia, dysthymia)	Unclear, no information	Out-patient	Cross-over, multi-arm	6 weeks	16^c^	Carbamazepine	Placebo	Depressive symptoms Anxiety symptoms
Crawford et al^56^	UK	75.36	36.1	Unclear, there is no information on population comorbidity. However, many psychiatric disorders were exclusion criteria	Mood stabilisers were not allowed, no further information	In- and out-patients	Parallel	52 weeks	276	Lamotrigine plus usual care	Placebo plus usual care	Depressive symptoms
De la Fuente and Lotstra^57^	Belgium	70	32.7	Unclear, no information	Not allowed	In-patient	Parallel	4 weeks	20	Carbamazepine	Placebo	Depressive symptoms Anxiety symptoms Psychotic symptoms
Frankenburg and Zanarini^58^	USA	100	26.85	Bipolar disorder type 2	Psychotropics not allowed	Out-patient	Parallel	24 weeks	30	Valproate semisodium	Placebo	Depressive symptoms
Hollander et al^59^	USA	52.38	38.6	Unclear, no information	Unclear, no information	Out-patient	Parallel	10 weeks	21^a^	Valproate semisodium	Placebo	Depressive symptoms
Loew et al^60^	Germany	100	25.25	Unclear, no information	Psychotropics not allowed	Out-patient	Parallel	10 weeks	56	Topiramate	Placebo	Depressive symptoms Anxiety symptoms Psychotic symptoms
Antidepressants versus placebo												
Cowdry and Gardner^46^	USA	100	31.6	Other Axis 2 personality disorders (dependent, avoidant, histrionic schizotypal, narcissistic and paranoid), and Axis 1 disorders (atypical bipolar disorder, major depressive episode with and without melancholia, dysthymia)	Unclear, no information	Out-patient	Cross-over, multi-arm	6 weeks	16^c^	Tranylcypromine sulfate	Placebo	Depressive symptoms Anxiety symptoms
Salzman et al^61^	USA	63.64^d^	36.3	Unclear, no information	Psychotropics not allowed	Out-patient	Parallel	12 weeks	22	Fluoxetine	Placebo	Depressive symptoms
Simpson et al^62^	USA	100	36.26	Comorbid Axis I pathology. No further information	Trazodone 50–100 mg/d for insomnia	In- and out-patient	Parallel	12 weeks	25	Fluoxetine plus DBT	Placebo plus DBT	Anxiety symptoms Psychotic symptoms
Soloff et al^53^	USA	75.56	25.1	Comorbid schizotypal personality disorder^^^^	Biperiden hydrochloride (2 mg)	In-patient	Parallel, multi-arm	5 weeks	90	Amitriptyline (haloperidol)	Placebo	Anxiety Depression Dissociation/psychosis
Cornelius et al^54^	USA	75.93	26.7	Major depressive disorder	Not allowed	In- and out-patients	Parallel, multi-arm	5 weeks	108	Phenelzine sulphate (haloperidol)	Placebo	Depression Anxiety Dissociation/psychosis

SCID-II, Structured Clinical Interview for DSM personality disorders; BPD, borderline personality disorder; SASPD, Standardised Assessment of Severity of Personality Disorder; ADHD, attention-deficit hyperactivity disorder; DBT, dialectical behavioural therapy; CNS, central nervous system; PTSD, post-traumatic stress disorder; ^^, inclusion criteria to enter the trial.

a. Initially, 21 participants entered the trial, and only 16 were randomised to a treatment group. No explanation for this was reported.

b. Trial had three arms with an overall sample size of 451, the sample size reported in this table is for the olanzapine 5–10 mg/d arm and placebo arm only.

c. Only included in secondary sensitivity analyses (cross-over trial with only end-of-period data available).

d. Completers only.

The trials were published between 1986 and 2024. Two were cross-over trials,^46,55^ and the rest had a parallel design. The smallest trial consisted of 16 participants,^46^ and the largest provided data on 314 participants.^65^ Four trials included more than 100 participants.^56,65–67^ Twelve trials were conducted in the USA, and eight were carried out in Europe. The remaining two multicentre trials included study sites mainly in North America and Europe.^65,66^

As within the 2022 Cochrane review, the placebo-controlled effect estimates available for meta-analyses were categorised into the three broad therapeutic classes: antipsychotics (including trials on aripiprazole, brexpiprazole, clozapine, haloperidol, olanzapine, quetiapine, trifluoperazine and ziprasidone),^28,44–49,51,53,55,63–67^ anticonvulsants (including carbamazepine, lamotrigine, topiramate, valproate)^46,57–60,68^ and antidepressants (amitriptyline, fluoxetine, phenelzine, tranylcypromine).^46,53,61,62,67^ Three trials^49,62,64^ had a concomitant dialectical behavioural therapy intervention in both arms, and one^68^ had concomitant treatment as usual in both arms (see main characteristics of trials that went into quantitative analyses, detailed in Table 1).

### Assessment of co-occurring psychopathology in the primary studies

In general, end-point data on symptoms of co-occurring psychopathology were sparsely investigated (Table 1). The outcome most reported on by the primary studies was depression. Thirteen trials of antipsychotics^44,46–49,51,53,55,63–67^ reported on this outcome, plus all included six trials of anticonvulsants^57–60,68,69^ and all five trials of antidepressants.^46,53,61,62,67^ Psychotic/dissociative symptoms were reported on by nine trials testing antipsychotics,^28,45,51,53,55,63,66,67,70^ three trials of anticonvulsants^57,60,68^ and three trials of antidepressants.^53,62,67^ The third outcome for which analyses could be done was anxiety, which was reported on by six antipsychotic trials,^46,51,53,63,64,67^ three anticonvulsant studies^46,57,60^ and four antidepressant trials.^46,53,62,67^ An overview of the measurement scales used to assess outcomes in the primary studies is available in Supplementary Appendix 5.

Although other outcomes of co-occurring psychopathology have been assessed by eligible primary studies, i.e. obsessive–compulsive disorder (OCD) symptoms^53,55,63,70^ substance use,^45,68^ schizotypal symptoms,^53,67^ bipolar disorder symptoms^44^ and PTSD,^55^ meta-analyses could not be conducted for any of them because of sparse data per intervention and outcome that would not have allowed for pooling at least three individual effect estimates.

### Risk of bias in included trials

None of trials included in the quantitative analysis had an overall low risk of bias. Fourteen trials^28,44–46,48,49,51,58,59,62,64–66,68^ had a high risk of bias in at least one domain, and the remaining had an unclear risk of bias in at least three domains.^28,53,57,60,61,63,67,70^

Random sequence generation was assessed to be of low risk of bias in eight trials,^28,48,49,51,58,65,66,68^ whereas the remaining trials did not describe their randomisation procedure sufficiently to permit a judgement of low or high risk, and for this reason, they were classed as of unclear risk of bias. Four trials^28,48,58,68^ were assessed to be of low risk of bias regarding allocation concealment. The remaining trials were of unclear risk of bias in this domain because insufficient reporting did not allow for a judgement of low or high risk of bias. Blinding of participants and personnel were assessed to be of low risk of bias in nine trials,^44–46,48,58,63,67,68,70^ high in one trial^28^ and the remaining trials in this domain were of unclear risk of bias. Blinding of outcome assessment was of low risk of bias in nine trials,^49,53,55,57,59,61,62,67,68^ high in one trial^28^ and all remaining trials in this domain were of unclear risk of bias. Incomplete outcome data was assessed as low risk of bias in five trials,^28,53,63,67,70^ and high risk of bias in eight trials.^44,46,48,51,55,59,62,68^ The remaining trials in this domain were assessed as unclear risk of bias. Similarly, for selective reporting, four trials were assessed as having a high risk of bias^28,44,65,71^ whereas the majority of the remaining trials had an unclear risk of bias. Eleven of the trials were assessed to be at high risk of bias because of vested interests (i.e. author affiliated with or funding of trials by pharmaceutical companies,^44,45,48,49,51,52,58,59,62,65,66^ and the majority of the remaining trials were assessed to be of unclear risk of bias in this regard. There seemed to be no indication of any other potential source of bias for the majority trials. One trial^46^ was rated as unclear in this domain because of an obvious carry-over effect between medication phases, and one trial^28^ was rated as having a high risk of bias because of the impact the COVID-19 pandemic had on the trial (early termination owing to severe problems recruiting participants). A risk-of-bias summary and graph are available in Supplementary Appendix 6.

### General treatment effects of major psychopharmacological classes on co-occurring psychopathology

#### Pharmacotherapy effects on co-occurring depressive symptoms

**Fig. 2:**
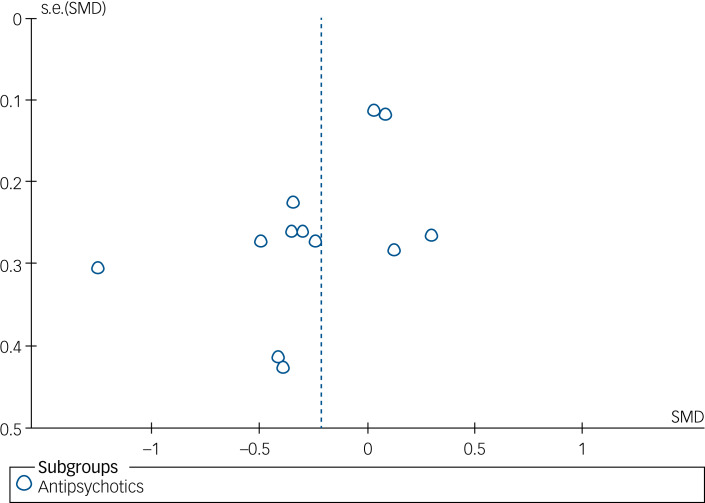
Funnel plot of included trials with antipsychotic intervention on depression at the end of treatment. SMD, standardised mean difference.

Twelve trials^44,46,48,49,51,53,63,64–67,70^ comparing antipsychotics and placebo had end-point data for depressive symptoms. There was evidence of a difference in depressive symptoms at the end of treatment (SMD −0.22, 95% CI −0.42 to −0.01, *P* = 0.04, 12 trials, *I*^2^ = 59%, very low certainty), favouring antipsychotics (Table 2). Upon visual inspection of the funnel plot, publication bias was strongly suspected in terms of a lack of studies reporting unfavourable effects (Fig. 2). The exclusion of the outlying study effect estimate of Nickel et al^63^ (SMD −1.25, 95% CI −1.85 to −0.65, 52 participants) resulted in a considerably smaller pooled effect estimate and less statistical heterogeneity (SMD −0.09, 95% CI −0.24 to 0.05, *P* = 0.26, *I*^2^ = 19%, 1066 participants).

**Table 2:** Effects of medications on co-occurring depressive, anxiety and dissociative-psychotic symptoms – summary of findings

Outcomes and interventions	Trials	*n*	Standardised mean difference^a^	95% CI	*P-*value	*I*^2^	Certainty of evidence (GRADE)
Depressive symptoms							
Antipsychotics	12^44,46,48,49,51,53,63–65,67,70^	1138	−0.22	−0.42 to −0.01	0.04	59%	⊕⊝⊝⊝ Very low^b^^,^^c^
Anticonvulsants	6^46,56–60^	344	−0.44	−0.80 to −0.08	0.02	46%	⊕⊕⊝⊝ Low^b^^,^^d^
Antidepressants	5^46,53,61,62,67^	187	−0.37	.0.82 to 0.08	0.11	52%	⊕⊝⊝⊝Very low^d^^,^^e^^,^^f^
Dissociative–psychotic symptoms							
Antipsychotics	9^28,45,51,53,63,65–67,70^	936	−0.28	−0.49 to −0.08	0.007	49%	⊕⊕⊝⊝ Low^b^
Anticonvulsants	3^56,57,60^	270	−0.23	−0.66 to 0.20	0.30	51%	⊕⊝⊝⊝ Very low^b^^,^^f^^,^^d^
Antidepressants	3^53,62,67^	139	−0.22	−0.62 to 0.18	0.29	25%	⊕⊝⊝⊝ Very low^b^^,^^f^^,^^d^
Anxiety symptoms							
Antipsychotics	6^46,51,53,63,64,67^	309	−0.35	−0.72 to 0.02	0.06	61%	⊕⊕⊝⊝ Low^b^^,^^d^
Anticonvulsants	3^46,57,63^	104	−1.11	−1.60 to −0.62	<0.00001	24%	⊕⊝⊝⊝ Very low^b^^,^^d^^,^^f^
Antidepressants	4^46,53,62,67^	164	−0.23	−0.58 to 0.12	0.20	17%	⊕⊝⊝⊝ Very low^b^^,^^d^^,^^f^

Grading of Recommendations, Assessment, Development and Evaluations (GRADE) Working Group grades of evidence: ‘high certainty’, further research is very unlikely to change our confidence in the estimate of effect; moderate certainty’, further research is likely to have an important impact on our confidence in the estimate of effect and may change the estimate; ‘low certainty’, further research is very likely to have an important impact on our confidence in the estimate of effect and is likely to change the estimate; ‘very low certainty’, we are very uncertain about the estimate.

a. Pooled standardised mean differences; negative values indicate beneficial effects of the experimental treatment. The inverse variance method and random effect model were used for pooling of effect estimates.

b. Downgraded two levels because of the inclusion of studies with a high risk of bias (i.e. several domains with a high risk of bias or most domains with unclear risk of bias).

c. Downgraded one level because of strongly suspected publication bias (cf. Fig. 2).

d. Publication bias undetected; unable to draw a funnel plot because of too few primary studies (≤10).

e. Downgraded two levels because of the inclusion of studies with a high risk of bias (i.e. several domains with a high risk of bias or most domains with unclear risk of bias).

f. Downgraded two levels because of a small sample size of <50% of optimal information size (assumed as *n* ≥ 400).

Five trials^46,53,61,62,67^ comparing antidepressants with placebo had end-point data for depressive symptoms. There was no evidence of a difference in depressive symptoms at the end of treatment between antidepressants and placebo (SMD −0.37, 95% CI −0.82 to 0.08, *P* = 0.11, five trials, *I*^2^ = 52%, very low certainty) (Table 2).

Six trials^46,57–60,68^ comparing anticonvulsants and placebo had end-point data for depressive symptoms. There was evidence of a difference in depression at the end of treatment, favouring anticonvulsants (SMD −0.44, 95% CI −0.80 to −0.08, *P* = 0.02, six trials, *I*^2^ = 46%, low certainty) (Table 2).

#### Pharmacotherapy effects on co-occurring anxious symptoms (post hoc)

Ten trials comparing antipsychotics and placebo had end-point data for anxious symptoms.^41,45,46,48,51,53,63,64,67,70^ However, only six of them^46,51,53,63,64,67^ reported usable data for generating summary estimates. Overall, there was no evidence of a difference between antipsychotics and placebo on anxious symptoms at the end of treatment (SMD −0.35, 95% CI −0.72 to 0.02, *I*^2^ = 61%, *P* = 0.06, six trials, 309 participants, very low certainty).

Six trials comparing antidepressants and placebo had end-point data for anxious symptoms.^42,43,46,53,62,67^ Unfortunately, two of them^42,43^ did not report usable data for generating summary estimates.

There was no evidence of a difference between antidepressants and placebo on anxiety (SMD −0.23, 95% CI −0.58 to 0.12, *I*^2^ = 17%, *P* = 0.20, four trials, 164 participants, very low certainty) (Table 2).

Three trials^46,57,60^ comparing anticonvulsants and placebo had end-point data for anxious symptoms. The summary estimate indicates evidence of a difference between anticonvulsants and placebo on anxiety, favouring anticonvulsants (SMD −1.11, 95% CI −1.60 to −0.62, *I*^2^ = 24%, *P* < 0.00001, three trials, 104 participants, very low certainty) (Table 2)

#### Pharmacotherapy effects on co-occurring dissociative symptoms

Nine trials^28,45,51,53,63,65–67,70^ comparing antipsychotics with placebo had end-point data for dissociative symptoms. There was evidence of a difference between groups at the end of treatment, favouring antipsychotics (SMD −0.28, 95% CI −0.49 to −0.08, *I*^2^ = 49%, nine trials, *P* = 0.007, 936 participants, low certainty).

Three trials^53,62,67^ with antidepressant interventions reported end-of-treatment data for dissociative symptoms. There was no evidence of a difference between antidepressants and placebo on dissociative symptoms at the end of treatment (SMD −0.22, 95% CI −0.62 to 0.18, *I*^2^ = 25%, *P* = 0.29, three trials, 139 participants, very low certainty) (Table 2). Only three trials^57,60,68^ reported dissociative symptoms for anticonvulsants at the end of treatment, and there was no evidence of a difference between groups (SMD −0.23, 95% CI −0.66 to 0.20, *I*^2^ = 51%, *P* = 0.30, three trials, 270 participants, very low certainty) (Table 2).

### Treatment effects of therapeutic classes: the role of co-occurring psychopathology

#### Treatment effects on depressive symptoms in trials including or excluding individual co-occurring psychopathology

Subgroup analyses were done for trials excluding versus not excluding PTSD, OCD, depression, bipolar disorder or psychotic disorders (Table 3, Supplementary Appendix 7). No statistically significant subgroup differences were observed for any of these analyses. However, data indicated a borderline significant subgroup differences (*P* = 0.05) between subgroups of trials that excluded participants with PTSD and those that did not: a numerically larger treatment effect was observed in the trials that excluded PTSD (SMD −0.32, 95% CI −0.60 to −0.03) versus those that did not exclude PTSD (SMD 0.02, 95% CI −0.15 to 0.18). The same was the case for subgroups of trials that excluded participants with OCD versus those that did not (test for subgroup differences*: P* = 0.05): numerically larger effects were reported for antipsychotics in trials that excluded OCD (SMD −0.32, 95% CI −0.60 to −0.03) versus those that did not exclude OCD (SMD 0.02, 95% CI −0.15 to 0.18).

**Table 3:** Differential effects of medications on co-occurring depressive, anxiety and dissociative–psychotic symptoms in relation to psychiatric exclusion criteria – summary of findings

Outcomes and interventions	Trials	*n*	Standardised mean difference^a^	95% CI	*P-*value	*I*^2^	Certainty of evidence (GRADE)	*χ*^2^-Test for subgroup differences *(p)*^b^
Antipsychotics: depression								
Substance use excluded	6^45,51,65–67,70^	799	−0.16	−0.30 to −0.02	0.02	0%	⊕⊕⊝⊝ Low^c^^,^^d^	0.001
Substance use not excluded	3^28,53,63^	137	−0.79	−1.14 to −0.44	<0.0001	0%	⊕⊝⊝⊝ Very low^d^^,^^e^^,^^f^	

Grading of Recommendations, Assessment, Development and Evaluations (GRADE) Working Group grades of evidence: ‘high certainty’, further research is very unlikely to change our confidence in the estimate of effect; ‘moderate certainty’, further research is likely to have an important impact on our confidence in the estimate of effect and may change the estimate; ‘low certainty’, further research is very likely to have an important impact on our confidence in the estimate of effect and is likely to change the estimate; ‘very low certainty’, we are very uncertain about the estimate.

a. Pooled standardised mean differences; negative values indicate beneficial effects of the experimental treatment. The inverse variance method and random effect model were used for pooling of effect estimates.

b. When including study data from the cross-over study of Ziegenhorn et al^55^ in a predefined secondary sensitivity analyses (see Stoffers-Winterling et al^12^), the *χ*^2^-test for subgroup differences reached statistical significance (*P* = 0.04) for the outcome of depression, if trials excluding (four trials, *N* = 473, SMD −0.32, 95% CI −0.60 to −0.03, *P* = 0.03, *I*^2^ = 55%) versus not excluding obsessive–compulsive disorder (four trials, *n* = 683, SMD 0.02, 95% CI −0.13 to 0.17, *P* = 0.84, *I*^2^ = 0%) were compared. No substantial changes were observed if including Ziegenhorn et al with any of the other relevant comparisons in terms of pooled effect estimates changing the direction of effect or crossing boundaries of confidence intervals.

c. Downgraded two levels because of the inclusion of studies with a high risk of bias (i.e. several domains with a high risk of bias or most domains with unclear risk of bias).

d. Publication bias undetected; unable to draw a funnel plot because of too few primary studies (≤10).

e. Downgraded two levels because of a small sample size <50% of optimal information size (assumed as *n* ≥ 400).

f. Downgraded one level because of the inclusion of studies with moderate risk of bias (i.e. high risk of bias for a maximum of one domain and/or unclear risk of bias for several domains).

Excluding the outlying effect estimate of Nickel et al from the subgroup analyses comparing trials with and without PTSD or OCD exclusion resulted in substantially lower heterogeneity within the individual comparison groups (PTSD/OCD excluded; *I*^2^ = 1% instead of *I*^2^ = 55%), a smaller pooled effect estimate (SMD −0.20 instead of SMD −0.32) and diminishing of the borderline significant subgroup effect (*P* = 0.10 instead of *P* = 0.05). Similarly, excluding the outlying effect estimate of Nickel et al from the subgroup analysis regarding psychotic disorders resulted in less heterogeneity within study estimates from trials excluding psychotic disorders (*I*^2^ = 8% instead of *I*^2^ = 63%), and a smaller pooled effect estimate (SMD −0.09 instead of SMD 0.28). The test for subgroup differences remained not significant (*P* = 0.80 instead of *P* = 0.35).

For antidepressants, no subgroup analyses could be done, as the minimum number of effect estimates per analysis was not reached.

For anticonvulsants compared with placebo, one subgroup analysis on trials excluding or including participants with depression was possible. There was no evidence of a difference in effect on depression at the end of treatment between trials that included participants with depression and trials that specifically excluded participants with depression (Table 3).

#### Treatment effects on anxious symptoms in trials including or excluding individual co-occurring psychopathology

For antipsychotics compared with placebo, it was not possible to conduct subgroup analyses, since the minimum number of three studies per subgroups was not reached for any psychopathology category (Table 3).

It was also not possible to do subgroup analyses for antidepressants or anticonvulsants, as the minimum number of effect estimates per subgroup was not reached.

#### Treatment effects on dissociative symptoms in trials including or excluding individual co-occurring psychopathology

For antipsychotics compared with placebo, it was possible to conduct subgroup analyses by trials excluding versus not excluding substance use and depression, respectively. There was evidence of a difference in dissociative symptoms at the end of treatment between trials that excluded participants with substance use (SMD −0.16, 95% CI −0.30 to −0.02) and those that did not (SMD −0.79, 95% CI −1.14 to −0.44), with a much larger effect size in the trials that did not exclude participants with substance use (test for subgroup differences: *P* = 0.001) (Table 3). There was no evidence of a difference in dissociative symptoms at the end of treatment between studies excluding and not excluding depression (Table 3).

For antidepressants and anticonvulsants, no subgroup analyses could be done, as the minimum number of effect estimates per subgroup was not reached.

## Discussion

This systematic review aimed to assess the effects of pharmacological interventions on non-BPD-specific psychopathology, and whether people diagnosed with BPD and co-occurring mental health disorders might respond differently to medications.

First, we assessed the evidence of pharmacological interventions on symptoms of co-occurring disorders in people with BPD. We were able to investigate the effects of co-occurring depressive, anxious and psychotic symptoms. We found evidence of small effects of antipsychotics on depressive (very low certainty) and dissociative symptoms (low certainty). However, the effect of antidepressants was not robust to the exclusion of an outlying study effect estimate. Moderate to large effects were found for anticonvulsants regarding depressive and anxiety symptoms (very low certainty). No evidence of any effects of antidepressants was observed for any outcome (very low certainty). Our previous Cochrane review already failed to identify any effects of antidepressants on BPD pathology (including impulsivity, for which selective serotonin reuptake inhibitors are frequently used), and this review complements the findings with regard to non-BPD-specific psychopathology. Given antidepressants are still among the most prevalently administered medications for patients with BPD in clinical settings,^72,73^ the results of this review are of high relevance to clinical practice.

Second, we explored if different effects could be observed in persons with BPD plus distinct psychiatric comorbidities. Comparing effect estimates from samples excluding versus including different types of co-occurring psychiatric disorders, we did not find any differences in effect regarding PTSD, OCD, MDD, bipolar disorders or psychotic disorders. The only subgroup difference found related to SUDs: trials including participants with SUDs reported a significantly greater reduction in dissociative symptoms by antipsychotics than those samples excluding people with a co-existing SUD. In other terms, antipsychotics had better outcomes in samples including participants with dual diagnoses. Although only a tentative finding, it might be of interest, as this patient group is conventionally regarded as having an unfavourable prognosis and worse treatment outcomes than persons with either condition alone.^74^ This assumption, however, has already been challenged by the availability of tailored psychotherapies (e.g. dialectical behaviour therapy for substance use disorders) and long-term observations.^75^

### Strengths and limitations

To our knowledge, this is the first meta-analysis that systematically investigates the effects of pharmacological interventions with BPD plus co-occurring disorders. It also highlights a lack of evidence on co-occurring psychopathology, since studies usually focus on few outcomes. Guideline developers and clinicians should be aware of these evidence gaps. Robust methods were applied as within the main Cochrane review, including comprehensive literature searches, which were updated for this review in April 2024. Predefined methods were applied, and any *post hoc* modifications made clear (see also Supplementary Appendix 2). The exploratory finding of greater effects by antipsychotics in samples including persons with a diagnosis of SUD may stimulate further research.

This review has some limitations. Ideally, co-occurring psychopathology in participants of trial samples should be investigated in the confirmed presence of a given disorder, and not based on exclusion criteria of trials. Given that such trials are lacking, and the question of intervention effects of pharmacotherapies in people with BPD is of high clinical relevance, we used exclusion criteria of primary studies as a proxy of the co-occurrence of disorders. A more ideal way of investigating the co-occurrence of disorders in our BPD samples would be to conduct meta-analyses of individual participant data, in which there is a certainty of co-occurring disorders in each participant. Such analyses are planned and underway for psychotherapeutic interventions,^76^ but to our knowledge, none are planned for pharmacological interventions.

Overall, the certainty of the evidence was low to very low. The risk of bias in included trials was overall moderate to high. Most of the included trials had small sample sizes and, as a result, the information size in most of the meta-analyses was low. This is especially true for the subgroup analyses, and as such, results from these should be considered exploratory. Given the low overall information size and small samples included in the individual trials, the power to detect existing differences was very likely too small (type 2 error); on the other hand, a random inflation of type 1 errors (i.e. falsely detecting a difference where there is none) cannot be ruled out. It was only possible to investigate publication bias by funnel plot inspection in one analysis (Fig. 2), therefore publication bias in the remaining analyses cannot be ruled out. Moreover, some estimates had significant heterogeneity. Most analyses in this review were conducted *post hoc.* We included multiple outcome measures and did not conduct tests for multiplicity, so the result from this article should be considered exploratory and for hypothesis generation. The absence of reported 95% confidence intervals alongside our calculated *I*^2^ values should be acknowledged as a limitation. The interpretation of *I*^2^ estimates warrants caution, especially in meta-analyses comprising a limited number of events or trials. Nonetheless, we addressed some of the observed heterogeneity by following the recommendation outlined in the Cochrane Handbook, specifically by excluding outliers. This approach is consistent with established methodologies.^22^

In conclusion, although our Cochrane review on medications for treatment of BPD failed to demonstrate substantial effects, medications are nonetheless prevalently prescribed to people with BPD, oftentimes with the rationale to target co-occurring psychopathology. Indeed, persons with BPD usually present with psychiatric symptoms that are not specific to BPD (e.g. depressive or anxious symptoms), which may or may not necessarily be explained by another co-occurring psychiatric disorder. However, a systematic review of medication effects on such co-occurring psychopathology, and the role of manifest comorbidities for treatment effects, has, to our knowledge, not yet been performed. The findings of this review aim to enable clinicians and persons affected by BPD to make informed decisions on whether to use medications to treat co-occurring psychopathology.

Medication effects on co-occurring psychopathology (i.e. depressive, dissociative–psychotic and anxiety symptoms) were small (antipsychotics) to moderate (anticonvulsants) to moderate. Tentative evidence suggests that persons with dual diagnoses (i.e. having BPD plus any SUD) might benefit better from antipsychotics regarding dissociative–psychotic symptoms than persons with BPD only.

It is important not to take the absence of robust, supporting evidence of medication effects, or limited findings of differential treatment effects linked to comorbidities, as ‘proof’ that no such effects or differences existed. Our findings, however, based on the currently available evidence – which is of low to very low certainty only – do not support the standard use of pharmacological interventions in people with BPD to target co-occurring psychopathology.

Research is urgently needed to shed light on medication effects in persons with defined comorbidities, given the vast majority of persons with BPD is affected by psychic comorbidities,^77^ and these comorbidities are usually accounted for as the major reason for using medications.

## Supporting information

Pereira Ribeiro et al. supplementary material 1 Pereira Ribeiro et al. supplementary material

Pereira Ribeiro et al. supplementary material 2 Pereira Ribeiro et al. supplementary material

## Data Availability

The data supporting the findings of this study are available within the article and/or its Supplementary materials. More data for reproducing results or replicating procedures are available from the original Cochrane review and/or on request from the corresponding author, O.J.S.
