# Supplementary material for: Pharmacological interventions for co-occurring psychopathology in people with borderline personality disorder: secondary analysis of the Cochrane systematic review with meta-analyses
Source: Br J Psychiatry. 2024 Oct 21;226(4):226–37. doi: 10.1192/bjp.2024.172 (PMC12038388; doi:10.1192/bjp.2024.172)
Supplement: Pereira Ribeiro et al. supplementary material 1 — Pereira Ribeiro et al. supplementary material [file S0007125024001727sup001.docx]

**Supplement S1: PRISMA 2020 Checklist**


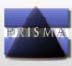
**PRISMA 2020 Checklist**

| **Section and Topic** | **Item #** | **Checklist item** | **Location where item is reported** |
| --- | --- | --- | --- |
| **TITLE** | | |  |
| Title | 1 | Identify the report as a systematic review. | p. 1 |
| **ABSTRACT** | | |  |
| Abstract | 2 | See the PRISMA 2020 for Abstracts checklist. | p. 3 |
| **INTRODUCTION** | | |  |
| Rationale | 3 | Describe the rationale for the review in the context of existing knowledge. | p. 3 |
| Objectives | 4 | Provide an explicit statement of the objective(s) or question(s) the review addresses. | p. 5 |
| **METHODS** | | |  |
| Eligibility criteria | 5 | Specify the inclusion and exclusion criteria for the review and how studies were grouped for the syntheses. | p. 5 |
| Information sources | 6 | Specify all databases, registers, websites, organisations, reference lists and other sources searched or consulted to identify studies. Specify the date when each source was last searched or consulted. | p. 5 |
| Search strategy | 7 | Present the full search strategies for all databases, registers and websites, including any filters and limits used. | p.5; Stoffers-Winterling et al., 2022^1^ |
| Selection process | 8 | Specify the methods used to decide whether a study met the inclusion criteria of the review, including how many reviewers screened each record and each report retrieved, whether they worked independently, and if applicable, details of automation tools used in the process. | pp. 5-6, Stoffers-Winterling et al., 2022^1^ |
| Data collection process | 9 | Specify the methods used to collect data from reports, including how many reviewers collected data from each report, whether they worked independently, any processes for obtaining or confirming data from study investigators, and if applicable, details of automation tools used in the process. | pp. 5-6, Stoffers-Winterling et al., 2022^1^ |
| Data items | 10a | List and define all outcomes for which data were sought. Specify whether all results that were compatible with each outcome domain in each study were sought (e.g. for all measures, time points, analyses), and if not, the methods used to decide which results to collect. | 5-6, Stoffers-Winterling et al., 2022^1^ |
|  | 10b | List and define all other variables for which data were sought (e.g. participant and intervention characteristics, funding sources). Describe any assumptions made about any missing or unclear information. | 5-6, Stoffers-Winterling et al., 2022^1^ |
| Study risk of bias assessment | 11 | Specify the methods used to assess risk of bias in the included studies, including details of the tool(s) used, how many reviewers assessed each study and whether they worked independently, and if applicable, details of automation tools used in the process. | pp.5-6 |
| Effect measures | 12 | Specify for each outcome the effect measure(s) (e.g. risk ratio, mean difference) used in the synthesis or presentation of results. | p. 6 |
| Synthesis methods | 13a | Describe the processes used to decide which studies were eligible for each synthesis (e.g. tabulating the study intervention characteristics and comparing against the planned groups for each synthesis (item #5)). | p. 6 |
|  | 13b | Describe any methods required to prepare the data for presentation or synthesis, such as handling of missing summary statistics, or data conversions. | Online supplement 2 |
|  | 13c | Describe any methods used to tabulate or visually display results of individual studies and syntheses. | p. 6 |
|  | 13d | Describe any methods used to synthesize results and provide a rationale for the choice(s). If meta-analysis was performed, describe the model(s), method(s) to identify the presence and extent of statistical heterogeneity, and software package(s) used. | p. 6 |
|  | 13e | Describe any methods used to explore possible causes of heterogeneity among study results (e.g. subgroup analysis, meta-regression). | p. 6 |
|  | 13f | Describe any sensitivity analyses conducted to assess robustness of the synthesized results. | p. 6 |
| Reporting bias assessment | 14 | Describe any methods used to assess risk of bias due to missing results in a synthesis (arising from reporting biases). | p. 6 |
| Certainty assessment | 15 | Describe any methods used to assess certainty (or confidence) in the body of evidence for an outcome. | p. 6 |
| **RESULTS** | | |  |
| Study selection | 16a | Describe the results of the search and selection process, from the number of records identified in the search to the number of studies included in the review, ideally using a flow diagram. | p. 6; Fig. 1 |
|  | 16b | Cite studies that might appear to meet the inclusion criteria, but which were excluded, and explain why they were excluded. | p. 6; online supplement S4 |
| Study characteristics | 17 | Cite each included study and present its characteristics. | Tab. 1 |
| Risk of bias in studies | 18 | Present assessments of risk of bias for each included study. | online supplement S6 |
| Results of individual studies | 19 | For all outcomes, present, for each study: (a) summary statistics for each group (where appropriate) and (b) an effect estimate and its precision (e.g. confidence/credible interval), ideally using structured tables or plots. | pp. 6-10, Tab. 2, Tab. 3 |
| Results of syntheses | 20a | For each synthesis, briefly summarise the characteristics and risk of bias among contributing studies. | pp. 6-10, Tab.2, Tab. 3 |
|  | 20b | Present results of all statistical syntheses conducted. If meta-analysis was done, present for each the summary estimate and its precision (e.g. confidence/credible interval) and measures of statistical heterogeneity. If comparing groups, describe the direction of the effect. | pp. 6-10, Tab. 2, Tab. 3 |
|  | 20c | Present results of all investigations of possible causes of heterogeneity among study results. | p.8; p. 9. |
|  | 20d | Present results of all sensitivity analyses conducted to assess the robustness of the synthesized results. | p. |
| Reporting biases | 21 | Present assessments of risk of bias due to missing results (arising from reporting biases) for each synthesis assessed. | p. 8 |
| Certainty of evidence | 22 | Present assessments of certainty (or confidence) in the body of evidence for each outcome assessed. | pp. 6-10, Tab.2, Tab. 3 |
| **DISCUSSION** | | |  |
| Discussion | 23a | Provide a general interpretation of the results in the context of other evidence. | p. 10 |
|  | 23b | Discuss any limitations of the evidence included in the review. | p. 11 |
|  | 23c | Discuss any limitations of the review processes used. | p. 11 |
|  | 23d | Discuss implications of the results for practice, policy, and future research. | pp. 11-12 |
| **OTHER INFORMATION** | | |  |
| Registration and protocol | 24a | Provide registration information for the review, including register name and registration number, or state that the review was not registered. | p. 5 |
|  | 24b | Indicate where the review protocol can be accessed, or state that a protocol was not prepared. | p. 5 |
|  | 24c | Describe and explain any amendments to information provided at registration or in the protocol. | pp. 4-6, online supplement S2 |
| Support | 25 | Describe sources of financial or non-financial support for the review, and the role of the funders or sponsors in the review. | p. 12 |
| Competing interests | 26 | Declare any competing interests of review authors. | p. 12 |
| Availability of data, code and other materials | 27 | Report which of the following are publicly available and where they can be found: template data collection forms; data extracted from included studies; data used for all analyses; analytic code; any other materials used in the review. | s. online Supplements S5; remaining materials are available upon request |

**^1^** Stoffers-Winterling, J. M., Storebø, O. J., Pereira Ribeiro, J., Kongerslev, M. T., Völlm, B. A., Mattivi, J. T., Faltinsen, E., Todorovac, A., Jørgensen, M. S., Callesen, H. E., Sales, C. P., Schaug, J. P., Simonsen, E., & Lieb, K. (2022). Pharmacological interventions for people with borderline personality disorder. *The Cochrane database of systematic reviews*, *11*(11), CD012956. https://doi.org/10.1002/14651858.CD012956.pub2

*From:*  Page MJ, McKenzie JE, Bossuyt PM, Boutron I, Hoffmann TC, Mulrow CD, et al. The PRISMA 2020 statement: an updated guideline for reporting systematic reviews. BMJ 2021;372:n71. doi: 10.1136/bmj.n71

**Supplement S2: Pre-defined methods not used in this review:**

- If all trials included in a meta-analysis of continuous data would have used the same measurement scale for assessing the outcome, we would have calculated mean differences (MDs) and corresponding 95% confidence intervals (95% CI).
  - We did not calculate any MDs as outcomes had been assessed by use of diverse outcome scales at all analyses.
- For dichotomous outcomes, we would have calculated the risk ratio and corresponding 95% CI with Mantel-Haenszel.
  - However, none of the primary studies of this review reported relevant dichotomous data.”
- In case of symmetrical funnel plots, we would subsequently have done an Egger’s test to investigate further on publication bias
  - The only funnel plot we were able to draw (because of a sufficient number of individual study effect estimates, i.e. ten or more) already indicated publication bias by asymmetry, therefore, an Egger’s test was not necessary.
- If the number of studies available would have permitted, we would have done subgroup analyses with studies of high/low risk of bias (minimum 3 effect estimates per subgroup).
  - We did not perform such subgroup analyses as there were too few studies to build up such subgroups.

| **Supplement S3:** Characteristics of eligible trials that were not available for quantitative analyses, with reasons | | | | | | | | | | | | |
| --- | --- | --- | --- | --- | --- | --- | --- | --- | --- | --- | --- | --- |
| **Study ID** | **Country** | **Sex (% female)** | **Age (years) Mean** | **Sample population Comorbidity** | **Concomitant medication allowed** | **Setting** | **Design** | **Duration** | **Sample size** | **Intervention** | **Comparator** | **reason for excludion from quantitative analyses** |
| Amminger 2013^1^ | Austria | 93.33 | 16.2 | Unclear. No information. | Antidepressants and benzodiazepines were allowed | Outpatient | Parallel | 12 weeks | 15 | Omega-3 fatty acids | Placebo | Insufficient number of poolable effect estimates per analyses |
| Hallahan 2007^2^ | Ireland | 65.31 | 30.6 | Unclear, no information. | Psychotropics as prescribed (if prescribed more than 6 weeks before screening) | Outpatient | Parallel | 12 weeks | 49 | Omega-3 fatty acids | Placebo | Insufficient number of poolable effect estimates per analyses |
| Maoz 2024 ^3^ | Israel | 88.57 | 29.94 | MDD^a^ | Yes | Inpatient | Parallel | 4 weeks | 35 | Intranasal oxytocin | Placebo | Insufficient number of poolable effect estimates per analyses |
| Markovitz 1995a^4^ | US | - | - | Each patient had on average 3 current Axis I diagnoses. A large proportion had comorbid personality disorders besides borderline: 82% self-defeating, 82% paranoid, 71% compulsive, 65% avoidant, 65% dependent, 59% histrionic, 59% passive-aggressive, 53% schizotypal, 35% narcissistic, 35% antisocial. | Unclear, no information. | Inpatient | Parallel | 14 weeks | 17 | Fluoxetine | Placebo | Data reported but unusable for effect size calculation |
| Moen 2012^5^ | US | 80 | 35.5 | Major depression | Not allowed | Inpatient | Parallel | 12 weeks | 15 | Valproate semisodium | Placebo | No relevant outcomes reported |
| Montgomery 1982a^6^ | UK | 70 | 35.05 | Unclear, no information | Unclear, no information | Outpatient | Parallel | 6 months | 30 | Flupentixol | Placebo | No relevant outcomes reported |
| Montgomery 1982b^6^ | UK | 68.42 | 35.65 | Unclear, no information | Unclear, no information | Outpatient | Parallel | 6 months | 28 | Mianserin | Placebo | No relevant outcomes reported |
| NCT00533117^7, b^ | US | 77.3 | 30.2 | Unclear, no information | Benzodiazepines were permitted for sleep | Inpatient | Parallel | 12 months | 37 ^d^ | Fluoxetine + DBT | Placebo + DBT | No relevant outcomes reported |
| NCT04566601 ^8^ | international (North and South Americas, Europe, Australia) | 86.2 | 30.2 | Current paranoid, schizoid, schizotypal or antisocial PD; lifetime schizophrenia, schizoaffective disorder, schizophreniform disorder, bipolar I disorder, or delusional disorder | Psychotropics not allowed | Outpatient | Prallel | 12 weeks | 390 | BI 1358894 | Placebo | Insufficient number of poolable effect estimates per analyses |
| Nickel 2004^9^ | Germany | 100 | 26.05 | Unclear, no information | Not allowed | Outpatient | Parallel | 8 weeks | 31 | Topiramate | Placebo | No relevant outcomes reported |
| Nickel 2005^10^ | Germany | 0 | 29.1 | mood disorders, somatoform disorders, anxiety disorders and eating disorders | Psychotropics not allowed | Outpatient | Parallel | 8 weeks | 44 | Topiramate | Placebo | No relevant outcomes reported |
| Kulkarni 2018^114^ | Australia | 85.29 | 34.4 | Bipolar II disorder | Antipsychotics, Antidepressants, and mood stabilisers allowed (TAU) | Outpatient | Parallel | 8 weeks | 34 | Memantine hydrochloride + TAU | Placebo +TAU | Insufficient number of poolable effect estimates per analyses |
| Reich 2009 ^12^*^)^* | US | 88.89 | 31.2 | major depression, PTSD, OCD, GAD, panic disorder, social phobia and specific phobia. | Patients could be taking one type of antidepressant but had to have been on a stable dose of that medication for 1 month. | Outpatient | Parallel | 12 weeks | 27 | Lamotrigine | Placebo | No relevant outcomes reported |
| Rinne 2002 ^13^ | The Netherlands | 100 | 29.2 | Depression, dysthymia, GAD, PTSD | Not allowed | Outpatient | Parallel | 6 weeks | 38 | Fluvoxamine | Placebo | Data reported but unusable for effect size calculation |
| Schmahl 2012a^14^ | Germany | 100 | 29.2 | Unclear, no information | Psychotropics not allowed | In- and outpatient | Cross-over | 6 weeks | 16 | Naltrexone 50mg | Placebo | Insufficient number of poolable effect estimates per analyses |
| Schmahl 2012b^14^ | Germany | 100 | 29.2 | pre-existing substance misuse | Psychotropics not allowed | In- and outpatient | Cross-over | 6 weeks | 16 | Naltrexone 200 mg | Placebo | Insufficient number of poolable effect estimates per analyses |
| Tritt 2005 ^15^ | Germany | 100 | 29.15 | Unclear, no information | Psychotropics not allowed | Outpatient | Parallel | 8 weeks | 27 | Lamotrigine | Placebo | No relevant outcomes reported |
| Zanarini 2001 ^16^ | US | 100 | 26.7 | Unclear, no information | Psychotropics not allowed | Outpatient | Parallel | 24 weeks | 28 | Olanzapine | Placebo | Data reported but unusable for effect size calculation |
| Zanarini 2003 ^17^ | US | 100 | 26.3 | Unclear, no information | Psychotropic medication or taking E-EPA supplements | Outpatient | Parallel | 8 weeks | 30 | Omega-3 fatty acids | Placebo (mineral oil) | Insufficient number of poolable effect estimates per analyses |
| DBT: Dialectical Behavioural Therapy; DSM: Diagnostic and Statistical Manual of Mental Disorders; E-EPA: ethyl-eicosapentaenoic acid; GAD: Generalised anxiety disorder; OCD: Obsessive-compulsive disorder; PD: Personality disorder; PTSD: Post-traumatic stress disorder  ^a^ Defined comorbidities according to inclusion criteria  ^b^Trial had 4 arms with an overall sample size of 86. The sample size reported in this table is for the fluoxetine+DBT arm and Placebo+DBT arm only.  ^¤^ Major depressive disorder without psychosis was a defined comorbidity for inclusion | | | | | | | | | | | | |

**References**

1 Amminger GP, Schäfer MR, Papageorgiou K, Klier CM, Cotton SM, Harrigan M SM, *et al.* Long-chain ω-3 fatty acids for indicated prevention of psychotic disorders: A randomized, placebo-controlled trial. *Arch Gen Psychiatry* 2010; **67**: 146–54.

2 Hallahan B, Hibbeln JR, Davis JM, Garland MR. Omega-3 fatty acid supplementation in patients with recurrent self-harm: Single-centre double-blind randomised controlled trial. *Br J Psychiatry* 2007; **190**: 118–22.

3 Maoz H, Grossman-Giron A, Sedoff O, Nitzan U, Kashua H, Yarmishin M, *et al.* Intranasal oxytocin as an adjunct treatment among patients with severe major depression with and without comorbid borderline personality disorder. *J Affect Disord* 2024; **347**: 39–44.

4 Markovitz P. Pharmacotherapy of impulsivity, aggression, and related disorders. In *Impulsivity and aggression* (ed SDJ Hollander E ): 263–87. Wiley, 1995.

5 Moen R, Freitag M, Miller M, Lee S, Romine A, Song S. Efficacy of extended-release divalproex combined with ‘condensed’ dialectical behavior therapy for individuals with borderline personality disorder. *Ann Clin Psychiatry* 2012; **24**: 255–60.

6 Montgomery SA, Montgomery D. Pharmacological prevention of suicidal behaviour. *J Affect Disord* 1982; **4**: 291–8.

7 NCT00533117. Treating Suicidal Behavior and Self-Mutilation in People With Borderline Personality Disorder. *https://clinicaltrials.gov/show/NCT00533117* 2007. (clinicaltrials.gov/ct2/show/results/NCT00533117?sect=X043716&amp;view=results#part).

8 Boehringer Ingelheim. A Study to Test Different Doses of BI 1358894 and Find Out Whether They Reduce Symptoms in People With Borderline Personality Disorder. clinicaltrials.gov. 2024. (https://clinicaltrials.gov/study/NCT04566601?a=14).

9 Nickel MK, Nickel C, Mitterlehner FO, Tritt K, Lahmann C, Leiberich PK, *et al.* Topiramate treatment of aggression in female borderline personality disorder patients: A double-blind, placebo-controlled study. *J Clin Psychiatry* 2004; **65**: 1515–9.

10 Nickel MK, Nickel C, Kaplan P, Lahmann C, Mühlbacher M, Tritt K, *et al.* Treatment of aggression with topiramate in male borderline patients: A double-blind, placebo-controlled study. *Biol Psychiatry* 2005; **57**: 495–9.

11 Kulkarni J, Thomas N, Hudaib AR, Gavrilidis E, Grigg J, Tan R, *et al.* Effect of the glutamate NMDA receptor antagonist memantine as adjunctive treatment in borderline personality disorder: an exploratory, randomised, double-blind, placebo-controlled trial. *Cent Nerv Syst Drugs* 2018; **32**: 179–87.

12 Reich DB, Zanarini MC, Bieri KA. A preliminary study of lamotrigine in the treatment of affective instability in borderline personality disorder. *Int Clin Psychopharmacol* 2009; **24**: 270–5.

13 Rinne T, Van den Brink W, Wouters L, Van Dyck R. SSRI treatment of borderline personality disorder: A randomized, placebo-controlled clinical trial for female patients with borderline personality disorder. *Am J Psychiatry* 2002; **159**: 2048–54.

14 Schmahl C, Kleindienst N, Limberger M, Ludäscher P, Mauchnik J, Deibler P, *et al.* Evaluation of naltrexone for dissociative symptoms in borderline personality disorder. *Int Clin Psychopharmacol* 2012; **27**: 61–8.

15 Tritt K, Nickel C, Lahmann C, Leiberich PK, Rother WK, Loew TH, *et al.* Lamotrigine treatment of aggression in female borderline-patients: A randomized, double-blind, placebo-controlled study. *J Psychopharmacol* 2005; **19**: 287–91.

16 Zanarini MC, Frankenburg FR, Parachini EA. A preliminary, randomized trial of fluoxetine, olanzapine, and the olanzapine-fluoxetine combination in women with borderline personality disorder. *J Clin Psychiatry* 2004; **65**: 903–7.

17 Zanarini MC, Ed D, Frankenburg FR. Omega-3 Fatty Acid Treatment of Women With Borderline Personality Disorder : *Am J Psychiatry* 2003; **160**: 167–9.

**Supplement S4: Reference list of trials included into quantitative analyses**

Black, D. W., Zanarini, M. C., Romine, A., Shaw, M., Allen, J., & Schulz, S. C. C. (2014). Comparison of low and moderate dosages of extended-release quetiapine in borderline personality disorder: a randomized, double-blind, placebo-controlled trial. *American Journal of Psychiatry*, *171*(11), 1174–1182. https://doi.org/10.1176/appi.ajp.2014.13101348

Bogenschutz, M. P., & Nurnberg, H. G. (2004). Olanzapine versus placebo in the treatment of borderline personality disorder. *Journal of Clinical Psychiatry*, *65*(1), 104–109. https://doi.org/10.4088/JCP.v65n0118

Cowdry, R. W., & Gardner, D. L. (1988). Pharmacotherapy of Borderline Personality Disorder: Alprazolam, Carbamazepine, Trifluoperazine, and Tranylcypromine. *Archives of General Psychiatry*, *45*(2), 111–119. https://doi.org/10.1001/archpsyc.1988.01800260015002

Crawford, M. J., Leeson, V. C., Evans, R., Barrett, B., McQuaid, A., Cheshire, J., Sanatinia, R., Lamph, G., Sen, P., Anagnostakis, K., Millard, L., Qurashi, I., Larkin, F., Husain, N., Moran, P., Barnes, T. R. E., Paton, C., Hoare, Z., Picchioni, M., & Gibbon, S. (2022). The clinical effectiveness and cost effectiveness of clozapine for inpatients with severe borderline personality disorder (CALMED study): a randomised placebo-controlled trial. *Therapeutic Advances in Psychopharmacology*, *12*, 204512532210908. https://doi.org/10.1177/20451253221090832

Crawford, M. J., Sanatinia, R., Barrett, B., Cunningham, G., Dale, O., Ganguli, P., Lawrence-Smith, G., Leeson, V., Lemonsky, F., Lykomitrou, G., Montgomery, A. A., Morriss, R., Munjiza, J., Paton, C., Skorodzien, I., Singh, V., Tan, W., Tyrer, P., & Reilly, J. G. (2018). The clinical effectiveness and cost-effectiveness of lamotrigine in borderline personality disorder: a randomized placebo-controlled trial. *American Journal of Psychiatry*, *175*(8), 756–764. https://doi.org/10.1176/appi.ajp.2018.17091006

De la Fuente, J., & Lotstra, F. (1994). A trial of carbamazepine in borderline personality disorder. *European Neuropsychopharmacology*, *4*(4), 479–486. https://doi.org/10.1016/0924-977X(94)90296-8

Frankenburg, F. R., & Zanarini, M. C. (2002). Divalproex sodium treatment of women with borderline personality disorder and bipolar II disorder: A double-blind placebo-controlled pilot study. *Journal of Clinical Psychiatry*, *63*(5), 442–446. https://doi.org/10.4088/JCP.v63n0511

Goldberg, C. S., Schulz, S. C., Schulz, M. P., Resnick, J. R., Hamer, M. R., Friedel, O. R., Goldberg, S. C., Schulz, S. C., Schulz, P. M., Resnick, R. J., Hamer, R. M., & Friedel, R. O. (1986). Borderline and schizotypal personality disorders treated with low-dose thiothixene vs placebo. *Archives of General Psychiatry*, *43*(7), 680–686.

Grant, J. E., Valle, S., Chesivoir, E., Ehsan, D., & Chamberlain, S. R. (2022). A double-blind placebo-controlled study of brexpiprazole for the treatment of borderline personality disorder. *British Journal of Psychiatry*, *220*(2), 58–63. https://doi.org/10.1192/bjp.2021.159

Hollander, E., Allen, A., Lopez, R. P., Bienstock, C. A., Grossman, R., Siever, L. J., Merkatz, L., Stein, D. J., & et, a. l. (2001). A preliminary double-blind, placebo-controlled trial of divalproex sodium in borderline personality disorder. *Journal of Clinical Psychiatry*, *62*(3), 199–203. https://doi.org/10.4088/JCP.v62n0311

Linehan, M. M., McDavid, J. P., Brown, M. Z., Sayrs, J. H. R., & Gallop, R. J. (2008). Olanzapine plus dialectical behavior therapy for women with high irritability who Meet criteria for borderline personality disorder: A double-blind, placebo-controlled pilot study. *Journal of Clinical Psychiatry*, *69*(6), 999–1005. https://doi.org/10.4088/JCP.v69n0617

Loew, T. H., Nickel, M. K., Muehlbacher, M., Kaplan, P., Nickel, C., Kettler, C., Fartacek, R., Lahmann, C., Buschmann, W., Tritt, K., Bachler, E., Mitterlehner, F., Gil, F. P., Leiberich, P., Rother, W. K., & Egger, C. (2006). Topiramate treatment for women with borderline personality disorder: A double-blind, placebo-controlled study. *Journal of Clinical Psychopharmacology*, *26*(1), 61–66. https://doi.org/10.1097/01.jcp.0000195113.61291.48

Nickel, M. K., Muehlbacher, M., Nickel, C., Kettler, C., Gil, F. P., Bachler, E., Buschmann, W., Rother, N., Fartacek, R., Egger, C., Anvar, J., Rother, W. K., Loew, T. H., & Kaplan, P. (2006). Aripiprazole in the treatment of patients with borderline personality disorder: A double-blind, placebo-controlled study. *American Journal of Psychiatry*, *163*(5), 833–838. https://doi.org/10.1176/ajp.2006.163.5.833

Pascual, J. C., Soler, J., Puigdemont, D., Pérez-Egea, R., Tiana, T., Alvarez, E., & Pérez, V. (2008). Ziprasidone in the treatment of borderline personality disorder: A double-blind, placebo-controlled, randomized study. *Journal of Clinical Psychiatry*, *69*(4), 603–608. https://doi.org/10.4088/JCP.v69n0412

Salzman, C., Wolfson, A. N., Schatzberg, A., Looper, J., Henke, R., Albanese, M., Schwartz, J., & Miyawaki, E. (1995). Effect of fluoxetine on anger in symptomatic volunteers with borderline personality disorder. *Journal of Clinical Psychopharmacology*, *15*(1), 23–29. https://doi.org/10.1097/00004714-199502000-00005

Schulz, S. C., Zanarini, M. C., Bateman, A., Bohus, M., Detke, H. C., Trzaskoma, Q., Tanaka, Y., Lin, D., Deberdt, W., & Corya, S. (2008). Olanzapine for the treatment of borderline personality disorder: Variable dose 12-week randomised double-blind placebo-controlled study. *British Journal of Psychiatry*, *193*(6), 485–492. https://doi.org/10.1192/bjp.bp.107.037903

Simpson, E. B., Yen, S., Costello, E., Rosen, K., Begin, A., Pistorello, J., & Pearlstein, T. (2004). Combined dialectical behavior therapy and fluoxetine in the treatment of borderline personality disorder. *Journal of Clinical Psychiatry*, *65*(3), 379–385. https://doi.org/10.4088/JCP.v65n0314

Soler, J., Pascual, J. C., Campins, J., Barrachina, J., Puigdemont, D., Alvarez, E., & Pérez, V. (2005). Double-blind, placebo-controlled study of dialectical behavior therapy plus olanzapine for borderline personality disorder. *American Journal of Psychiatry*, *162*(6), 1221–1224. https://doi.org/10.1176/appi.ajp.162.6.1221

Soloff, P. H., Cornelius, J., George, A., Nathan, S., Perel, J. M., & Ulrich, R. F. (1993). Efficacy of Phenelzine and Haloperidol in Borderline Personality Disorder. *Archives of General Psychiatry*, *50*(5), 377–385. https://doi.org/10.1001/archpsyc.1993.01820170055007

Soloff, P. H., George, A., Nathan, R. S., Schulz, P. M., Cornelius, J. R., Herring, J., & Perel, J. M. (1989). Amitriptyline versus haloperidol in borderlines: Final outcomes and predictors of response. *Journal of Clinical Psychopharmacology*, *9*(4), 238–246. https://doi.org/10.1097/00004714-198908000-00002

Zanarini, M.C., Schulz, S. C., Detke, H. C., Tanaka, Y., Zhao, F., Lin, D., DeBerdt, W., & Corya, S. (2007). A dose comparison of olanzapine for the treatment of borderline personality disorder: A 12-week randomized double-blind placebo-controlled study. *European Psychiatry*, *22*(Suppl 1), S172–S173. https://doi.org/10.1016/j.eurpsy.2007.01.565

Ziegenhorn AA, Roepke S, Schommer NC, Merkl A, Danker-Hopfe H, Perschel FH, *et al.* (2009). Clonidine improves hyperarousal in borderline personality disorder with or without comorbid posttraumatic stress disorder: a randomized, double-blind, placebo-controlled trial. *J Clin Psychopharmacol*; 29, 170–3.

| **Supplement S5: Measurement scales of primary studies included into quantitative analyses** | | | |
| --- | --- | --- | --- |
| **Study ID** | **Measurement scale** | | |
|  | **Depression** | **Anxiety** | **Dissociation/psychosis** |
| Black 2014 | Montgomery-Åsberg Depression Rating Scale | - | - |
| Bogenschutz 2004 | - | Hamilton Anxiety Rating Scale^¤^ | CGI-BPD (transient paranoia or dissociation) |
| Cowdry 1988 | CGI or modified Bunny Hamburg scale* | Modified Bunney-Hamburg Scale Clinician | - |
| Crawford 2018  Crawford 2022 | Beck Depression Inventory  - | -  - | ZAN-BPD-Cognitive disturbance  BPRS |
| De la Fuente 1994 | Hamilton Depression Rating Scale (24 items) | SCL-90 anxiety subscale | SCL-90 R psychoticism |
| Frankenburg 2002 | SCL-90-R Depression | - | - |
| Goldberg 1986 | Hopkins Symptoms check List - Depression | Hopkins Symptom Checklist 90^¤^ | SIB-Suspicious/paranoid |
| Grant 2022 | Hamilton Depression Rating Scale | Hamilton Anxiety Rating Scale^¤^ | - |
| Hollander 2001 | Beck Depression Inventory | - | - |
| Linehan 2008 | Hamilton Depression Rating Scale | - | - |
| Loew 2006 | SCL-90-R Depression | SCL-90 anxiety subscale | SCL-90 R psychoticism |
| Nickel 2006 | Hamilton Depression Rating Scale | Hamilton Anxiety Rating Scale | SCL-90 R psychoticism |
| Pascual 2008 | Hamilton Depression Rating Scale (17 items) | Hamilton Anxiety Rating Scale | CGI-BPD (transient paranoia or dissociation) |
| Salzman 1995 | Hamilton Depression Rating Scale | - | - |
| Schulz 2007 | Montgomery-Åsberg Depression Rating Scale | - | ZAN-BPD-paranoid ideation |
| Simpson 2004 | Beck Depression Inventory | State-Trait Anxiety Inventory | Dissociative Experiences Scale |
| Soler 2005 | Hamilton Depression Rating Scale | Hamilton Anxiety Rating Scale | - |
| Soloff 1989 | Beck Depression Inventory | SCL-90 anxiety subscale | SCL-90 R psychoticism |
| Soloff 1993 | Beck Depression Inventory | SCL-90 anxiety subscale | SCL-90 R psychoticism |
| Zanarini 2007  Ziegenhorn 2009 | Montgomery-Åsberg Depression Rating Scale  Beck Depression Inventory | -  - | ZAN-BPD-paranoid ideation  - |
| BPRS: Brief Psychiatric Rating Scale; CGI-BPD: Clinical Global Impression scale for Borderline Personality Disorder; SCL-90-R: Symptom Check List-90 – Revised; SIB: Schedule of Interviewing Schizotypal Personalities - Borderline score; ZAN-BPD: Zanarini rating scale for borderline personality disorder.  ** “At the end of each trial, we obtained ratings of clinical change on seven-point scales similar in concept to the Clinical Global Improvement scale, on which the patient or physician was asked to rate change on each scale "compared to a usual month prior to the start of the study.” (Cowdry 1988, p. 11).*  *¤ Unable to generate summary estimates of reported data.* | | | |

**Supplement S6: Risk of bias graph and summary**


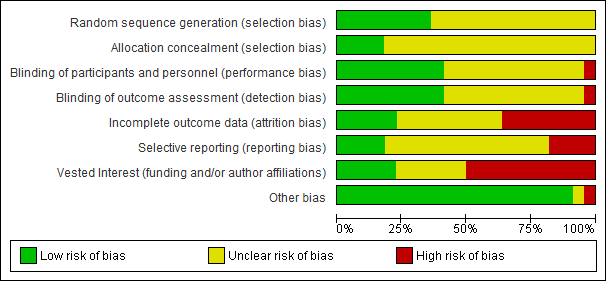


Figure S6.1

Risk of bias graph: review authors' judgements about each risk of bias item presented as percentages across all included studies


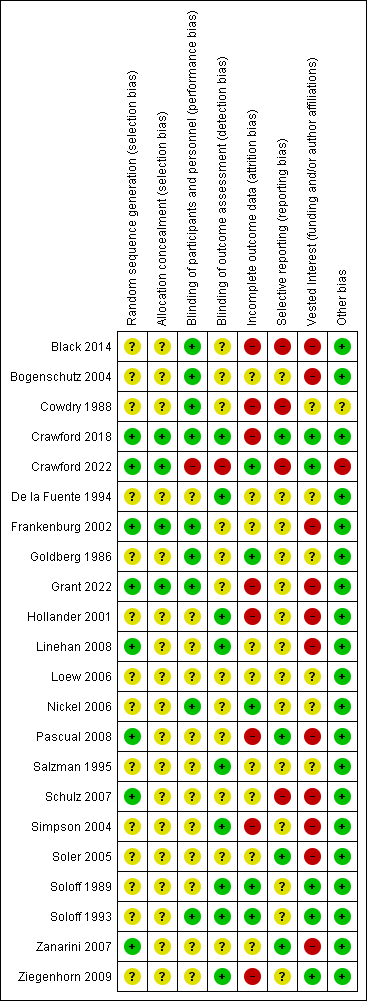


Figure S6.2

Risk of bias summary: review authors' judgements about each risk of bias item for each included trial

| **Supplement S7a: Effects of antipsychotics on co–occurring psychopathology overall and in subgroups of exclusion criteria for included trials.** | | | | | | | | | |
| --- | --- | --- | --- | --- | --- | --- | --- | --- | --- |
| **Outcomes and subgroups** | **Trials** | **N** | **Statistical**  **method** | **Effect size*** | **95% CI** | ***p*** | **I^2^** | **Certainty of evidence**  **(GRADE)** | **Chi^2^ Test for subgroup differences *(p)*** |
| Depressive symptoms | 12^1–12^ | 1138 | IV, random | SMD –0.22 | –0.42 to –0.01 | 0.04 | 59% | ⊕⊝⊝⊝ Very low^e,h^ |  |
| PTSD excluded | 9^1,2,5–11^ | 473 | IV, random | SMD –0.32 | –0.60 to –0.03 | 0.03 | 55% | ⊕⊕⊝⊝ Low^e^ | 0.05 |
| PTSD not excluded | 3^3,4,12^ | 665 | IV, random | SMD 0.02 | –0.15 to 0.18 | 0.85 | 13% | ⊕⊝⊝⊝ Very low^a,e^ |  |
| OCD excluded | 9^1,2,5–11^ | 473 | IV, random | SMD –0.32 | –0.60 to –0.03 | 0.03 | 55% | ⊕⊕⊝⊝ Low^e^ | 0.05^#^ |
| OCD not excluded | 3^3,4,12^ | 665 | IV, random | SMD 0.02 | –0.15 to 0.18 | 0.85 | 13% | ⊕⊝⊝⊝ Very low^a,e^ |  |
| Depression excluded | 6^2,6–8,10,11,13^ | 268 | IV, random | SMD –0.40 | –0.84 to 0.04 | 0.07 | 67% | ⊕⊝⊝⊝ Very low^b,e,g^ | 0.14 |
| Depression not excluded | 6^1,3–5,9,12^ | 870 | IV, random | SMD –0.05 | –0.21 to 0.11 | 0.54 | 18% | ⊕⊝⊝⊝ Very low^a,e^ |  |
| Bipolar disorder excluded | 9^2–7,9,10,12^ | 933 | IV, random | SMD –0.08 | –0.23 to 0.06 | 0.27 | 11% | ⊕⊕⊝⊝ Low^e^ | 0.42 |
| Bipolar disorder not excluded | 3^1,8,10^ | 205 | IV, random | SMD –0.42 | –1.22 to 0.39 | 0.31 | 86% | ⊕⊝⊝⊝ Very low^d,e,g^ |  |
| Psychotic disorders excluded | 9^1–4,6–9,12^ | 974 | IV, random | SMD -0.28 | ­–0.52 to –0.04 | 0.02 | 63% | ⊕⊝⊝⊝ Very low^e,g^ | 0.35 |
| Psychotic disorders not excluded | 3^5,10,11^ | 164 | IV, random | SMD -0.02 | –0.50, 0.45 | 0.09 | 58% | ⊕⊝⊝⊝ Very low^a, f^ |  |
| Anxiety symptoms | 6^2,3,8–11^ | 309 | IV, random | SMD –0.35 | –0.72 to 0.02 | 0.06 | 61% | ⊕⊕⊝⊝ Very low^e^ |  |
| Dissociative symptoms | 9^4,5,8–12,14,15^ | 936 | IV, random | SMD –0.28 | –0.49 to –0.08 | 0.007 | 49% | ⊕⊕⊝⊝ Low^e^ |  |
| Substance use excluded | 6^4,5,9,11,12,14^ | 799 | IV, random | SMD –0.16 | –0.30 to –0.02 | 0.02 | 0% | ⊕⊕⊝⊝ Low^e^ | 0.001 |
| Substance use not excluded | 3^8,10,15^ | 137 | IV, random | SMD –0.79 | –1.14 to –0.44 | < 0.0001 | 0% | ⊕⊝⊝⊝ Very low^c,f^ |  |
| Depression excluded | 5^4,5,9,12,14^ | 741 | IV, random | SMD –0.16 | –0.31 to –0.02 | 0.03 | 0% | ⊕⊕⊝⊝ Low^e^ | 0.07 |
| Depression not excluded | 4^8,10,11,15^ | 195 | IV, random | SMD –0.59 | –1.01 to –0.16 | 0.007 | 53% | ⊕⊝⊝⊝ Very low^c,f,g^ |  |
| *Negative MDs or SMDs and RRs >1 indicate beneficial effects of the experimental treatment  IV: Inversed variance; M–H: Mantel-Haenszel; N: Total number of participants; OCD: Obsessive-compulsive disorder; PTSD: Post-traumatic stress disorder; SMD: Standardised mean difference  ^#^When including study data from the cross-over study of Ziegenhorn et al. (2009) in a pre-defined secondary sensitivity analyses (s. Cochrane Review^16^, the Chi^2^ Test for subgroup differences reaches statistical significance (*p*=0.04). No substantial changes were observed if including Ziegenhorn et al. (2009) to any of the other relevant comparisons in terms of pooled effect estimates changing the direction of effect or crossing boundaries of confidence intervals.  a downgraded 2 levels due to trivial difference or no difference, with the possibility of difference in both directions  b downgraded 1 level due to a small sample size representing less than 70% but more than 50% of optimal information size (assumed as n≥400)  c downgraded 2 levels due to a small sample size less than 50% of optimal information size (assumed as n≥400)  d downgraded 3 levels due to a small sample size plus CI boundaries suggest very different inferences  e downgraded 2 levels due to the inclusion of studies with a high risk of bias (i.e., several domains with a high risk of bias or most domains with unclear risk of bias)  f downgraded 1 level due to the inclusion of studies with moderate risk of bias (i.e., high risk of bias for a maximum of one domain and/or unclear risk of bias for several domains)  g downgraded 1 level due to substantial heterogeneity (unexplained statistical heterogeneity not explained by clinical or methodological heterogeneity); χ2 p values <.05 (if certainty had been downgraded for imprecision already due to small sample size, we downgraded only 1 level for inconsistency, even if considerable statistical heterogeneity was present)  h downgraded 1 level due to high risk of publication bias (cf. Fig. 4)  GRADE Working Group grades of evidence High certainty: Further research is very unlikely to change our confidence in the estimate of effect. Moderate certainty: Further research is likely to have an important impact on our confidence in the estimate of effect and may change the estimate.  Low certainty: Further research is very likely to have an important impact on our confidence in the estimate of effect and is likely to change the estimate.  Very low certainty: We are very uncertain about the estimate. | | | | | | | | | |

| **Supplement S7b: Effects of antidepressants on co–occurring depression, anxiety and dissociation.** | | | | | | | | | |
| --- | --- | --- | --- | --- | --- | --- | --- | --- | --- |
| **Outcomes and subgroups** | **Trials** | **N** | **Statistical**  **method** | **Effect size*** | **95% CI** | ***p*** | **I^2^** | **Certainty of evidence**  **(GRADE)** | **Chi^2^ Test for subgroup differences *(p)*** |
| Depressive symptoms | 5^2,10,11,17,18^ | 187 | IV, random | SMD –0.37 | –0.82 to 0.08 | 0.11 | 52% | ⊕⊝⊝⊝ Very low^a,b^ |  |
| Anxiety symptoms | 4^2,10,11,18^ | 164 | IV, random | SMD –0.23 | –0.58 to 0.12 | 0.20 | 17% | ⊕⊝⊝⊝ Very low^a,b^ |  |
| Dissociative symptoms | 3^10,11,18^ | 139 | IV, random | SMD –0.22 | –0.62 to 0.18 | 0.29 | 25% | ⊕⊝⊝⊝ Very low^a,b^ |  |
| *Negative MDs or SMDs and RRs >1 indicate beneficial effects by the experimental treatment; IV: Inversed variance; M–H: Mantel-Haenszel; N: Total number of participants SMD: Standardised mean difference  a downgraded 2 levels due to a small sample size of less than 50% of optimal information size (assumed as n≥400)  b downgraded 2 levels due to the inclusion of studies with a high risk of bias (i.e., several domains with a high risk of bias or most domains with unclear risk of bias)  GRADE Working Group grades of evidence High certainty: Further research is very unlikely to change our confidence in the estimate of effect. Moderate certainty: Further research is likely to have an important impact on our confidence in the estimate of effect and may change the estimate.  Low certainty: Further research is very likely to have an important impact on our confidence in the estimate of effect and is likely to change the estimate.  Very low certainty: We are very uncertain about the estimate. | | | | | | | | | |

| **Supplement S7c. Effects of anticonvulsants on co–occurring depression, anxiety and dissociation overall and in subgroups of exclusion criteria for included trials.** | | | | | | | | | |
| --- | --- | --- | --- | --- | --- | --- | --- | --- | --- |
| **Outcomes and subgroups** | **Trials** | **N** | **Statistical**  **method** | **Effect size*** | **95% CI** | ***p*** | **I^2^** | **Certainty of evidence**  **(GRADE)** | **Chi^2^ test for subgroup differences (*p*)** |
| Depressive symptoms | 6^2,19–23^ | 344 | IV, random | SMD –0.44 | –0.80 to –0.08 | 0.02 | 46% | ⊕⊕⊝⊝  Low^b^ |  |
| Depression excluded | 3^19–21^ | 65 | IV, random | SMD –0.66 | –1.19 to –0.12 | 0.02 | 0% | ⊕⊝⊝⊝ Very low^a,b^ | 0.42 |
| Depression not excluded | 3^2,22,23^ | 279 | IV, random | SMD –0.36 | –0.86 to 0.15 | 0.16 | 66% | ⊕⊝⊝⊝ Very low^a,b^ |  |
| Anxiety symptoms | 3^2,8,19^ | 104 | IV, random | SMD –1.11 | –1.60 to –0.62 | <0.00001 | 24% | ⊕⊝⊝⊝ Very low^a,b^ |  |
| Dissociative symptoms | 3^19,22,23^ | 270 | IV, random | SMD –0.23 | –0.66 to 0.20 | 0.30 | 51% | ⊕⊝⊝⊝ Very low^a,b^ |  |
| *Negative MDs or SMDs and RRs >1 indicate beneficial effects of the experimental treatment  IV: Inversed variance; M–H: Mantel-Haenszel; N: Total number of participants SMD: Standardised mean difference  a downgraded 2 levels due to a small sample size of less than 50% of optimal information size (assumed as n≥400)  b downgraded 2 levels due to the inclusion of studies with a high risk of bias (i.e., several domains with a high risk of bias or most domains with unclear risk of bias)  GRADE Working Group grades of evidence High certainty: Further research is very unlikely to change our confidence in the estimate of effect. Moderate certainty: Further research is likely to have an important impact on our confidence in the estimate of effect and may change the estimate.  Low certainty: Further research is very likely to have an important impact on our confidence in the estimate of effect and is likely to change the estimate.  Very low certainty: We are very uncertain about the estimate. | | | | | | | | | |

**References**

1 Black DW, Zanarini MC, Romine A, Shaw M, Allen J, Schulz SCC. Comparison of low and moderate dosages of extended-release quetiapine in borderline personality disorder: a randomized, double-blind, placebo-controlled trial. *Am J Psychiatry* 2014; **171**: 1174–82.

2 Cowdry RW, Gardner DL. Pharmacotherapy of Borderline Personality Disorder: Alprazolam, Carbamazepine, Trifluoperazine, and Tranylcypromine. *Arch Gen Psychiatry* 1988; **45**: 111–9.

3 Soler J, Pascual JC, Campins J, Barrachina J, Puigdemont D, Alvarez E, *et al.* Double-blind, placebo-controlled study of dialectical behavior therapy plus olanzapine for borderline personality disorder. *Am J Psychiatry* 2005; **162**: 1221–4.

4 Schulz SC, Zanarini MC, Bateman A, Bohus M, Detke HC, Trzaskoma Q, *et al.* Olanzapine for the treatment of borderline personality disorder: Variable dose 12-week randomised double-blind placebo-controlled study. *Br J Psychiatry* 2008; **193**: 485–92.

5 Goldberg CS, Schulz SC, Schulz MP, Resnick JR, Hamer MR, Friedel OR, *et al.* Borderline and schizotypal personality disorders treated with low-dose thiothixene vs placebo. *Arch Gen Psychiatry* 1986; **43**: 680–6.

6 Grant JE, Valle S, Chesivoir E, Ehsan D, Chamberlain SR. A double-blind placebo-controlled study of brexpiprazole for the treatment of borderline personality disorder. *Br J Psychiatry* 2022; **220**: 58–63.

7 Linehan MM, McDavid JP, Brown MZ, Sayrs JHR, Gallop RJ. Olanzapine plus dialectical behavior therapy for women with high irritability who Meet criteria for borderline personality disorder: A double-blind, placebo-controlled pilot study. *J Clin Psychiatry* 2008; **69**: 999–1005.

8 Nickel MK, Muehlbacher M, Nickel C, Kettler C, Gil FP, Bachler E, *et al.* Aripiprazole in the treatment of patients with borderline personality disorder: A double-blind, placebo-controlled study. *Am J Psychiatry* 2006; **163**: 833–8.

9 Pascual JC, Soler J, Puigdemont D, Pérez-Egea R, Tiana T, Alvarez E, *et al.* Ziprasidone in the treatment of borderline personality disorder: A double-blind, placebo-controlled, randomized study. *J Clin Psychiatry* 2008; **69**: 603–8.

10 Soloff PH, George A, Nathan RS, Schulz PM, Cornelius JR, Herring J, *et al.* Amitriptyline versus haloperidol in borderlines: Final outcomes and predictors of response. *J Clin Psychopharmacol* 1989; **9**: 238–46.

11 Soloff PH, Cornelius J, George A, Nathan S, Perel JM, Ulrich RF. Efficacy of Phenelzine and Haloperidol in Borderline Personality Disorder. *Arch Gen Psychiatry* 1993; **50**: 377–85.

12 Zanarini MC, Schulz SC, Detke HC, Tanaka Y, Zhao F, Lin D, *et al.* A dose comparison of olanzapine for the treatment of borderline personality disorder: A 12-week randomized double-blind placebo-controlled study. *Eur Psychiatry* 2007; **22**: S172–3.

13 Ziegenhorn AA, Roepke S, Schommer NC, Merkl A, Danker-Hopfe H, Perschel FH, *et al.* Clonidine improves hyperarousal in borderline personality disorder with or without comorbid posttraumatic stress disorder: a randomized, double-blind, placebo-controlled trial. *J Clin Psychopharmacol* 2009; **29**: 170–3.

14 Bogenschutz MP, Nurnberg HG. Olanzapine versus placebo in the treatment of borderline personality disorder. *J Clin Psychiatry* 2004; **65**: 104–9.

15 Crawford MJ, Leeson VC, Evans R, Barrett B, McQuaid A, Cheshire J, *et al.* The clinical effectiveness and cost effectiveness of clozapine for inpatients with severe borderline personality disorder (CALMED study): a randomised placebo-controlled trial. *Ther Adv Psychopharmacol* 2022; **12**: 204512532210908.

16 Stoffers-Winterling JM, Storebø OJ, Pereira Ribeiro J, Kongerslev MT, Völlm BA, Mattivi JT, *et al.* Pharmacological interventions for people with borderline personality disorder. *Cochrane Database Syst Rev* 2022; **2022**. doi:10.1002/14651858.CD012956.pub2.

17 Salzman C, Wolfson AN, Schatzberg A, Looper J, Henke R, Albanese M, *et al.* Effect of fluoxetine on anger in symptomatic volunteers with borderline personality disorder. *J Clin Psychopharmacol* 1995; **15**: 23–9.

18 Simpson EB, Yen S, Costello E, Rosen K, Begin A, Pistorello J, *et al.* Combined dialectical behavior therapy and fluoxetine in the treatment of borderline personality disorder. *J Clin Psychiatry* 2004; **65**: 379–85.

19 De la Fuente J, Lotstra F. A trial of carbamazepine in borderline personality disorder. *Eur Neuropsychopharmacol* 1994; **4**: 479–86.

20 Frankenburg FR, Zanarini MC. Divalproex sodium treatment of women with borderline personality disorder and bipolar II disorder: A double-blind placebo-controlled pilot study. *J Clin Psychiatry* 2002; **63**: 442–6.

21 Hollander E, Allen A, Lopez RP, Bienstock CA, Grossman R, Siever LJ, *et al.* A preliminary double-blind, placebo-controlled trial of divalproex sodium in borderline personality disorder. *J Clin Psychiatry* 2001; **62**: 199–203.

22 Crawford MJ, Sanatinia R, Barrett B, Cunningham G, Dale O, Ganguli P, *et al.* The clinical effectiveness and cost-effectiveness of lamotrigine in borderline personality disorder: a randomized placebo-controlled trial. *Am J Psychiatry* 2018; **175**: 756–64.

23 Loew TH, Nickel MK, Muehlbacher M, Kaplan P, Nickel C, Kettler C, *et al.* Topiramate treatment for women with borderline personality disorder: A double-blind, placebo-controlled study. *J Clin Psychopharmacol* 2006; **26**: 61–6.
